# Loss of Smell in COVID-19 Patients: Lessons and Opportunities

**DOI:** 10.3389/fnhum.2020.598465

**Published:** 2020-11-26

**Authors:** Dennis Mathew

**Affiliations:** Department of Biology, University of Nevada, Reno, NV, United States

**Keywords:** SARS-CoV-2, COVID-19, olfaction, diabetes, ACE2, insulin

## Introduction

When Rebecca Medrano, a Washington D.C. resident who tested positive for SARS-CoV-2 infection, first started feeling unwell with viral symptoms, she noticed an additional symptom—she had lost her sense of smell (NPR.org, 2020). Worldwide, doctors have reported similar cases of smell and taste loss in up to 60% of COVID-19 patients, now confirmed in a peer-reviewed study (Yan et al., 2020). A second unexpected observation was that infected patients with diabetes had higher rates of serious complications and death (ADA, 2020; Barron et al., 2020).

Underlying mechanisms for these observations remain unclear. In addition, they raise important questions: Does SARS-CoV-2 infect olfactory tissue and if so, which olfactory cell types do they infect and how do viral mechanisms in these cells modulate smell function? Does diabetes exacerbate viral infection or does virus-mediated dysregulation of diabetic mechanisms further aggravate symptoms in diabetics? These are critical questions to focus on amidst the tremendous increase in research efforts related to this pandemic.

Within just few months, the number of SARS-CoV-2 cases has crossed 40 million with over one million deaths reported around the world (Johns Hopkins Coronavirus Resource Center, 2020). A major focus of current pandemic-related research efforts seems to be on virus entry and host immunity mechanisms, and on developing vaccines. While these are important research goals, unless we develop effective drugs that are disease specific, this virus is likely to persist and mutate causing more harm to human life and economies around the world. There is thus an urgent need for basic research into viral mechanisms and to generate innovative strategies to slow down disease progression, especially in diabetics.

The cases of Rebecca Medrano and many like her losing their sense of smell highlight unique opportunities to study SARS-CoV-2 mechanisms. Such an opportunity to better understand SARS-CoV-2 mechanisms and its relationship to diabetes might be found in our nose. Here, we argue that our communal fight against this dreaded disease would benefit greatly from an additional focus on studying viral mechanisms in olfactory tissue. Olfactory tissue offers a convenient venue to explore interactions between viral and diabetic mechanisms and evaluate them at the level of function (loss of smell).

### How Does SARS-CoV-2 Infection Lead to Smell Loss?

While many COVID-19 patients have reported a loss in their ability to smell, it is unclear how this virus mediates smell loss. Is there SARS-CoV-2 activity in olfactory tissue or could loss in smell simply be a byproduct of an overactive host immune response to the virus? Ample recent evidence supports the idea that viral mechanisms play a role in olfactory tissue.

SARS-CoV-2 uses the receptor, Angiotensin-converting enzyme 2 (ACE2) to attach to a host cell. ACE2, a metalloprotease is found in cells of several tissues including olfactory tissue (Hamming et al., 2004; Sungnak et al., 2020). Within olfactory tissue, ACE2 and TMPRSS2 (a priming protease that facilitates viral uptake) express in several cell types (Hamming et al., 2004; Baig et al., 2020; Bilinska et al., 2020; Brann et al., 2020; Sungnak et al., 2020). Expression of these genes in olfactory cell types suggests that olfactory tissue may be sensitive to SARS-CoV-2 infections. It may also explain why FDA approved ACE inhibitors such as Captopril are associated with loss in smell and taste (Doty and Bromley, 2004).

However, early studies are unresolved as to whether infection of olfactory sensory neurons (OSNs) or of neighboring cells is responsible for smell loss. One possibility is that smell loss may be due to death of OSNs (Netland et al., 2008; Baig et al., 2020). Immunostainings of SARS-Co-V N protein revealed high abundance of this antigen in olfactory bulb of infected mice. This study suggested SARS-virus entry via olfactory nerve with subsequent transneural spread into brain regions. The olfactory nerve, which consists mainly of OSNs and directly connects nasal cavity with the central brain, is considered a shortcut for several viruses including the influenza virus to gain entry into the brain (van Riel et al., 2015). Viral infections of olfactory nerve are usually accompanied by concomitant (non-apoptotic) neuronal death (Netland et al., 2008).

Another possibility is that infection of non-neural olfactory cells and not OSNs may be responsible (Bilinska et al., 2020; Brann et al., 2020). Bilinska et al. report high expression of ACE2 and TMPRSS2 in sustentacular cells of the olfactory epithelium and less expression in OSNs (Bilinska et al., 2020). Since sustentacular cells play key roles in supporting OSN metabolism and odor sensing, any virus-mediated damage to these cells could lead to olfactory deficits (Heydel et al., 2013). Brann et al. also report that ACE2 and TMPRSS genes are expressed in sustentacular cells, HBCs, microvillar cells, and Bowman's gland cells of the olfactory epithelium but not in OSNs. They argue that viral infection of sustentacular cells may be enough to cause a pathophysiological cascade that culminates in damage to OSNs and thereby cause smell loss. They also found ACE2 expression in vascular pericytes, which are involved in inflammatory response (Brown et al., 2019; Brann et al., 2020).

Overall, these studies offer several hypotheses as to how viral mechanisms could lead to smell loss in COVID-19 patients: *one* ACE2 mechanisms in olfactory neurons lead to a direct modulation of olfactory sensitivities or even cell death; *two* ACE2 mechanisms in non-neural olfactory cells lead to indirect modulation of OSN function and; *three* ACE2 mechanisms in neighboring glial cells lead to increased inflammatory response, whose downstream effects could alter OSN function and reduce olfactory sensitivities. While this is not an exhaustive list of possible hypotheses, it's a start. Each hypothesis needs to be rigorously assessed so that we may better understand why many COVID-19 patients lose their sense of smell.

### Why Do Diabetics Have Worse Outcomes After SARS-CoV-2 Infections?

SARS-CoV-2-infected patients with diabetes face higher rates of serious complications and even death (ADA, 2020). Type 1 and type 2 diabetics have up to 3.5 times the odds of dying with COVID-19 (Barron et al., 2020). A recent meta-analysis study of data collected from 1,527 patients in China revealed that diabetes was among the most prevalent comorbidities associated with COVID-19 (Li et al., 2020). These observations raise important questions: Could diabetes-associated symptoms aggravate viral infection? Conversely, could viral infection aggravate diabetes symptoms?

A relationship between various infections and diabetes has long been debated. Population-based studies have revealed that infections such as influenza and pneumonia are common and more serious among older people with type 2 diabetes (McDonald et al., 2014; Pearson-Stuttard et al., 2016; Li et al., 2019). While diabetes may predispose individuals to certain infections, it is less clear how (Knapp, 2013). There are several theories as to how diabetes and SARS-CoV-2 associate to influence symptom severity and mortality. A simple explanation is that high levels of inflammation, coagulation, immune response impairment, etc. in diabetics could aggravate viral infection and symptoms (Hussain et al., 2020).

However, there is also evidence to support a case for complex interactions between viral and diabetic mechanisms within cells. For instance, upon being fed a high calorie diet, ACE2 knockout mice had impaired glucose tolerance compared to their wild type littermates (Takeda et al., 2013). This result suggests that ACE2 expressed in insulin-sensitive tissues plays a critical role in maintaining glucose homeostasis and insulin sensitivity. Thus, a SARS-CoV-2/ACE2-mediated dysregulation of insulin signaling could further aggravate symptoms in diabetics.

### Searching for Insights in the Nose

The olfactory tissue offers a convenient venue to research interactions between viral and diabetic mechanisms and gain fundamental insights about SARS-CoV-2 mechanisms. As described above, several olfactory cell types express ACE2 and TMPRSS, likely rendering them sensitive to viral infections. It turns out that olfactory tissue is also sensitive to insulin. In fact, highest density of central insulin receptors and highest concentration of insulin in mammalian brain are found in the olfactory bulb (Havrankova et al., 1981). Insulin signaling plays a significant role in satiety-dependent modulation of smell sensitivities (Bargmann, 2012; Taghert and Nitabach, 2012; Ko et al., 2015; Slankster et al., 2020). Like ACE2, insulin receptors are expressed on surface of both OSNs and glia (Nassel et al., 2013; Musashe et al., 2016). Using the olfactory system, we could ask whether ACE2 and insulin mechanisms interact and, if they do, identify downstream molecules that link these mechanisms. Identifying downstream molecules that link ACE2 and insulin mechanisms will expand our understanding of how viral and diabetic mechanisms interact to impact cell function. Additionally, these results may inform studies in other tissues and generate drug targets with relevance for disease progression in diabetics.

Olfactory systems, especially in genetically tractable model organisms like mice and *Drosophila* may aid in our fight against this virus. These systems have been commonly used as convenient *in vivo* model systems for disease research (Steuer et al., 2014; Franks et al., 2015). Previous studies have characterized *Drosophila*-orthologs of human ACE (*AnCE* and *ACER*) (Cornell et al., 1995). Notably, the same drugs that inhibit human ACE also inhibit ACE orthologs in *Drosophila* (Kim et al., 2003). Genetic tools to manipulate ACE receptor and insulin signaling in mice and *Drosophila* olfactory cells have been described and are available in public collections. Considering olfactory tissue offers a convenient venue to explore interactions between viral and diabetic mechanisms as well as to evaluate them at the level of function, we cannot ignore the possibility that olfactory research will lead to insights that may aid our fight against this virus. Few other tissues harbor both ACE2 and insulin mechanisms and even when they do, such as in lungs and liver, studying functional interactions in these tissues can be challenging. We therefore urge research agencies to consider funding basic research focused on viral mechanisms in olfactory tissue.

## Discussion

SARS-CoV-2 has negatively impacted lives around the world. Amidst growing concerns of its impact, federal and private agencies are rushing to develop effective cures and vaccines. While there is a need for such solutions, there is also a need for basic research into underlying viral mechanisms and thereby generate more innovative solutions. One area that needs more basic research is the relationship between viral and diabetic mechanisms. Could abnormal insulin signaling in olfactory neurons of diabetics exacerbate SARS-CoV-2-mediated mechanisms and lead to loss in smell in Covid-19 patients? This relationship can no longer be ignored, especially since diabetics infected with SARS-CoV-2 have up to 3.5 times the odds of serious complications and death. Fortunately, stories of Rebecca Medrano and others offer us an opportunity to tackle this enemy from a different perspective and eventually defeat it. Studying interactions between viral and diabetic mechanisms in olfactory tissue of genetically tractable model organisms such as mice and *Drosophila* offers a different approach. Such an approach will not only lead to new insights about SARS-CoV-2 mechanisms but may also help identify potential drug targets primarily relevant to disease progression in diabetics. Identifying viable drug candidates will greatly impact our collective capability of fighting this disease over the next 5–10 years. If scientists and funding agencies consider novel but thoughtful actions right now, they will help change course of events and thus make a significant impact in the long term.

## Author Contributions

DM conceived and wrote this opinion piece.

## Conflict of Interest

The author declares that the research was conducted in the absence of any commercial or financial relationships that could be construed as a potential conflict of interest.
